# Commentary: Characterization of gut microbiota and metabolites in individuals with constipation-predominant irritable bowel syndrome

**DOI:** 10.3389/fmicb.2026.1726737

**Published:** 2026-01-30

**Authors:** Xuesong Tang, Yingjie Liu, Wenjiang Wu, Xingxing Tao

**Affiliations:** Shenzhen Hospital (Futian) of Guangzhou University of Chinese Medicine, Shenzhen, Guangdong, China

**Keywords:** constipation-predominant irritable bowel syndrome (IBS-C), gut microbiota, IBS, Principal Co-ordinates Analysis (PCoA), short-chain fatty acids (SCFAs)

## Introduction

The study by Wang et al. (2025) represents a timely contribution to the burgeoning field of gut microbiome research in functional gastrointestinal disorders, particularly constipation-predominant irritable bowel syndrome (IBS-C). By integrating metagenomic and metabolomic analyses, the authors successfully unravel the complex interplay between microbial dysbiosis and metabolic perturbations, offering new insights into IBS-C pathogenesis. Their findings of reduced beneficial bacteria like *Megasphaera elsdenii* and *Bifidobacterium bifidum*, alongside impaired short-chain fatty acid (SCFA) metabolism, align with the prevailing hypothesis that gut microbiota influences host physiology through metabolite production (Cani, 2018; Lynch and Pedersen, 2016). However, while the study adeptly identifies associative patterns, it also highlights several translational challenges and unresolved mechanistic gaps that warrant further investigation. This commentary seeks to contextualize these findings within the broader scientific discourse, highlighting both the strengths and limitations of the approach, and proposing directions for future research to bridge correlation with causation. The exploration of microbial metabolites not only deepens our understanding of IBS-C but also underscores the potential for developing microbiota-targeted therapies, provided the methodological and interpretive hurdles are adequately addressed.

## Methodological considerations and analytical depth

A primary strength of Wang et al.'s work lies in its multi-omics framework, which moves beyond descriptive microbiota profiling to incorporate functional metabolomic insights. The identification of differential metabolites enriched in pathways like phenylalanine metabolism and SCFA biosynthesis offers a plausible link between microbial composition and host symptoms such as reduced motility. For instance, the observed downregulation of butyrate—a key SCFA known to enhance colonic motility and maintain epithelial integrity—provides a tangible mechanistic clue. Although the original study was appropriately powered for its primary endpoints, the inherent inter-individual variability of the gut microbiome means that the generalizability of specific species-level findings (e.g., the reduction in *Aistipes inops*) must be confirmed in larger, more diverse cohorts. This would also allow for subgroup analyses based on different IBS-C phenotypes. Therefore, future studies with larger cohorts are imperative to account for the high inter-individual variability in gut microbiota and to validate these subtle metabolic shifts. While the correlation analyses are a valuable first step, the reliance on Spearman's rank correlation alone limits causal inference. As noted in the discussion, employing advanced integration methods like MaAsLin2 could help identify more robust, multivariate associations that are better candidates for future causal validation. Furthermore, while the identification of differentially abundant species such as *Megasphaera elsdenii* and *Aistipes inops* is informative, it is important to note that metagenomic sequencing often lacks the resolution to discern strain-level variations, which can exhibit significant functional heterogeneity. For instance, the observed reduction in *Bifidobacterium bifidum* abundance warrants deeper investigation, as different strains of this species possess distinct genomic arsenals for mucin degradation and carbohydrate fermentation. The putative functions inferred from KEGG pathway analysis (e.g., “starch and sucrose metabolism”) should be complemented with *in vitro* cultivation assays or targeted quantification of specific bacterial enzymes to confirm these metabolic activities are indeed impaired in IBS-C patients. Although the authors note a positive correlation between *M. elsdenii* and acetic acid levels, it remains unclear whether this relationship is driven by direct microbial metabolism or confounding host factors. Future studies could benefit from incorporating longitudinal sampling and interventional designs (e.g., fecal microbiota transplantation or probiotic supplementation) to test causal hypotheses (Mars et al., 2020). For instance, a recent FMT trial in IBS patients demonstrated the potential of such interventional approaches to elucidate causality (Halkjær et al., 2023). The acknowledged lack of dietary data, a common challenge in observational studies, is a critical confounder. Future interventional studies that control or manipulate dietary intake (e.g., providing standardized meals) are essential to isolate the specific contributions of the microbiota to IBS-C pathophysiology from dietary effects (Wark et al., 2020). This is particularly relevant in IBS, where dietary interventions such as the low-FODMAP diet are known to profoundly alter gut microbiota and symptom profiles (Molina-Infante et al., 2016; Staudacher and Whelan, 2017). Controlling for such confounders is critical to isolate the specific contributions of microbiota to IBS-C pathophysiology.

## Interpretation of findings and clinical implications

The study's most compelling findings revolve around the convergence of microbial dysbiosis and metabolic dysfunction, particularly the downregulation of SCFAs and upregulation of leukotriene D5 (LTD5), a pro-inflammatory eicosanoid. This dual observation supports a model where reduced SCFA production impairs gut motility and barrier function, while elevated LTD5 may promote low-grade inflammation—a hallmark of IBS. The authors rightly speculate that *B. bifidum* depletion could diminish mucin degradation and SCFA synthesis, but this remains hypothetical without experimental validation. Similarly, the putative role of LTD5 in increasing intestinal permeability is intriguing but remains speculative, as it is primarily extrapolated from studies on structurally similar molecules like LTD4. The convergence of microbial dysbiosis and metabolic dysfunction, particularly the downregulation of SCFAs and upregulation of pro-inflammatory mediators, supports a pathophysiological model of IBS-C. The enrichment of differential metabolites in phenylalanine metabolism is a particularly notable finding (Wang et al., 2025, Figure 6). Phenylalanine is a precursor for tyrosine, which in turn is a precursor for dopamine and norepinephrine. Alterations in this pathway may not only affect local gut physiology but also influence the gut-brain axis, potentially contributing to the abdominal discomfort and altered motility characteristic of IBS-C. To strengthen this link, future research could measure serum or fecal levels of key neurotransmitters or their precursors (e.g., tyrosine, homovanillic acid) to directly assess the functional output of this metabolic perturbation. To strengthen these claims, targeted assays measuring epithelial barrier integrity (e.g., transepithelial electrical resistance) or inflammatory markers (e.g., fecal calprotectin) in future work would be invaluable. From a clinical perspective, the findings hint at potential therapeutic avenues, such as probiotics targeting *Bifidobacterium* or prebiotics to boost SCFA production. However, the study's cross-sectional design precludes conclusions about the temporal dynamics of these alterations. For example, it is unclear whether dysbiosis precedes symptom onset or results from chronic constipation. Integrating multi-omics data with clinical phenotypes (e.g., stool frequency, pain scores) through machine learning models could help identify biomarker signatures predictive of disease progression or treatment response (Reel et al., 2021).

## Discussion

In summary, Wang et al. provide a valuable foundational dataset underscoring the role of gut microbiota and metabolites in IBS-C. Their integrated approach highlights the importance of moving beyond taxonomic profiling to functional analyses, yet the study also exemplifies the challenges inherent in human microbiome research—including sample size constraints, confounding variables, and correlative limitations. To advance the field, future studies should prioritize longitudinal designs, dietary harmonization, and mechanistic experiments to validate putative pathways (Zhao et al., 2020). By addressing these gaps, researchers can transform associative findings into actionable insights, ultimately paving the way for personalized microbiota-modulating therapies for IBS-C patients. Furthermore, while the multi-omics framework is a strength, the integration of metagenomic and metabolomic data remains primarily correlative. The study employs Spearman's rank correlation, which identifies associations but falls short of delineating directional interactions or causal pathways between specific microbial functions and metabolite alterations. To advance from correlation to causation, future studies could employ more advanced integration methods such as *Multivariate Association with Linear Models* (MaAsLin2) or *Sparse Partial Least Squares Discriminant Analysis* (sPLS-DA) (Mallick et al., 2021). The application of such multivariate frameworks has already yielded novel insights into microbiome-host interactions in other complex diseases, providing a template for IBS-C research (Franzosa et al., 2019). These techniques can help identify key microbial genes or pathways that directly predict metabolite levels, thereby constructing a more mechanistic model of gut microbiome-host interactions in IBS-C.
